# EEG Driven tDCS Versus Bifrontal tDCS for Tinnitus

**DOI:** 10.3389/fpsyt.2012.00084

**Published:** 2012-09-25

**Authors:** Dirk De Ridder, Sven Vanneste

**Affiliations:** ^1^Brai^2^n and Translational Neuroscience, University Hospital AntwerpAntwerp, Belgium

**Keywords:** tinnitus, EEG, tDCS, direct current, gamma

## Abstract

Tinnitus is the perception of a sound in the absence of any objective physical sound source. Transcranial Direct Current Stimulation (tDCS) induces shifts in membrane resting potentials depending on the polarity of the stimulation: under the anode gamma band activity increases, whereas under the cathode the opposite occurs. Both single and multiple sessions of tDCS over the dorsolateral prefrontal cortex (DLPFC; anode over right DLPFC) yield a transient improvement in tinnitus intensity and tinnitus distress. The question arises whether optimization of the tDCS protocol can be obtained by using EEG driven decisions on where to place anode and cathode. Using gamma band functional connectivity could be superior to gamma band activity as functional connectivity determines the tinnitus network in many aspects of chronic tinnitus. Six-hundred-seventy-five patients were included in the study: 265 patients received tDCS with cathodal electrode placed over the left DLPFC and the anode placed overlying the right DLPFC, 380 patients received tDCS based on EEG connectivity, and 65 received no tDCS (i.e., waiting list control group). Repeated measures ANOVA revealed a significant main effect for pre versus post measurement. Bifrontal tDCS in comparison to EEG driven tDCS had a larger reduction for both tinnitus distress and tinnitus intensity. Whereas the results of the bifrontal tDCS seem to confirm previous studies, the use of gamma band functional connectivity seems not to bring any advantage to tDCS for tinnitus suppression. Using other potential biomarkers, such as gamma band activity, or theta functional connectivity could theoretically be of use. Further studies will have to elucidate whether brain state based tDCS has any advantages over “blind” bifrontal stimulation.

## Introduction

Tinnitus is the perception of a sound or sounds (e.g., a tone, hissing, or buzzing sound, or a combination of such sounds) in the absence of any objective physical sound source (Jastreboff, 1990). In western societies about 5–15% of the population has chronic tinnitus and will seek medical attention (Axelsson and Ringdahl, 1989; Heller, 2003). Tinnitus often causes a considerable amount of distress: between 6 and 25% of the affected people report symptoms that are severely debilitating (Baguley, 2002; Eggermont and Roberts, 2004).

Based on functional imaging studies, including fMRI (Smits et al., 2007), EEG (van der Loo et al., 2009; Vanneste et al., 2010a), MEG (Muhlnickel et al., 1998; Weisz et al., 2007), and PET (Lockwood et al., 1999; Langguth et al., 2006) it is generally accepted that tinnitus is related to auditory cortex hyperactivity and maladaptive plasticity, often due to damage of the peripheral auditory system. But co-activation of non-auditory brain structures such as the insula (Smits et al., 2007; Vanneste et al., 2010a; van der Loo et al., 2011), anterior cingulate cortex (Muhlau et al., 2006; Plewnia et al., 2007; Rauschecker et al., 2010; Vanneste et al., 2010a; Leaver et al., 2011; Vanneste et al., 2011c), and dorsolateral prefrontal cortex (DLPFC; Schlee et al., 2009a; Vanneste et al., 2010a) has been described as well, and some pathophysiological mechanisms have been proposed based on these studies (Rauschecker et al., 2010; De Ridder et al., 2011a; Leaver et al., 2011). This has led to the concept that the unified tinnitus percept is the result of one large tinnitus network consisting of multiple dynamically adaptive overlapping subnetworks (De Ridder et al., 2011a), with each subnetwork representing a clinically separable aspect such as distress (Vanneste et al., 2010a; De Ridder et al., 2011b), sound characteristic (noise-like versus pure tone; Vanneste et al., 2010b), lateralization (Vanneste et al., 2011a), etc.

The DLPFC has an important function in auditory processing. Bilateral DLPFC has a facilitatory effect on auditory memory storage and contains auditory memory cells (Bodner et al., 1996). This prefrontal area also exerts early inhibitory modulation of input to primary auditory cortex in humans (Knight et al., 1989) and has been found to be associated with auditory attention (Alain et al., 1998; Lewis et al., 2000; Voisin et al., 2006) resulting in top-down modulation of auditory processing (Mitchell et al., 2005). This has been further confirmed by electrophysiological data indicating that tinnitus might occur as the result of a dysfunction in the top-down inhibitory processes (Norena et al., 1999; Faber et al., 2011).

Transcranial Direct Current Stimulation (tDCS) is an old neuromodulation tool which recently has seen a revival. In tDCS, a weak direct electrical current (1–2 mA) is applied to the scalp, through which most of the current is shunted. But about 50% of the transcranially applied direct current reaches the brain, both in animal models (Rush and Driscoll, 1968) and humans (Dymond et al., 1975). This current induces shifts in membrane resting potentials, thereby depolarizing or hyperpolarizing neurons (Nitsche et al., 2003) depending on the polarity of the stimulation. tDCS induces an increase or decrease in cortical excitability in the brain regions to which it is applied (Nitsche and Paulus, 2000; Miranda et al., 2006). Anodal tDCS typically has an excitatory effect on the local cortical excitability by inducing a relative neuronal depolarization, while cathode has an opposite effect – it induces a hyperpolarization (Nitsche and Paulus, 2001). tDCS was first applied to the auditory cortex in an attempt to improve tinnitus (Fregni et al., 2006), and does indeed seem to be able to induce long-lasting changes in tinnitus perception (Garin et al., 2011).

Based on the influence of the DLPFC on auditory processing and its involvement in tinnitus it was demonstrated that a single session of tDCS over the DLPFC (anode over right DLPFC) yields a transient improvement in both tinnitus intensity and tinnitus distress in subjects with chronic tinnitus (Vanneste et al., 2010c), where as stimulation with anode over left DLPFC induces no changes in tinnitus (Vanneste et al., 2010c). When applying repetitive sessions this could be proposed as a treatment (Faber et al., 2011; Frank et al., 2012). The efficacy of bifrontal tDCS for transient tinnitus suppression depends on the brain state (Vanneste et al., 2011b). Applying multiple sessions of bifrontal tDCS has been proposed as potential treatment for tinnitus (Frank et al., 2012). The question arises whether optimalization of the tDCS protocol can be obtained by using EEG driven decisions on where to place anode and cathode. Based on the pathophysiology of tinnitus and the polarity dependent effect of tDCS it can be proposed to place the (inhibitory) cathode at an area of tinnitus related gamma band activity (De Ridder et al., 2007, 2011a,c; Lorenz et al., 2009; Schlee et al., 2009b; van der Loo et al., 2009), or even better gamma band functional connectivity (Vanneste et al., 2011b; Schlee et al., 2009a). Using gamma band functional connectivity could be superior to gamma band activity as functional connectivity determines the tinnitus network in many aspects of chronic tinnitus (Vanneste et al., 2010b, 2011b,d; Schlee et al., 2009b; Vanneste and De Ridder, 2011).

## Methods and Materials

### Participants

Six-hundred-seventy-five subjects (260 males and 415 females) with chronic tinnitus (>1 year) were recruited from the Tinnitus Clinic at the University Hospital Antwerp, Belgium and participated in this retrospective study, with a mean age of 48.33 years (*Md* = 50; SD = 14.57). The mean tinnitus duration was 5.14 years (*Md* = 4; SD = 4.24). In order to obtain a homogeneous sample and exclude potential variables that would interfere with response to tDCS, we excluded subjects based on the following criteria: individuals with pulsatile tinnitus, a history of epileptic insults, severe organic co-morbidity, a pacemaker, or defibrillator, a present pregnancy, neurological disorders such as brain tumors, and individuals being treated for mental disorders. All prospective subjects underwent a complete ENT and neurological investigation to rule out possible treatable causes for their tinnitus. All patients younger than 18 years were excluded from the study. Table 1 further shows the tinnitus characteristics for both groups. The study was in accordance with the ethical standards of the Helsinki declaration (1964) and was approved by the institutional ethics committee of the Antwerp University Hospital.

**Table 1:** **Tinnitus characteristics**.

		Groups		
		Frontal tDCS	EEG driven tdcs	Waiting list
Mean duration		5.11	5.22	4.80
Type	Pure tone	85	134	21
	Narrow band noise	188	246	44
Laterality	Unilateral	148	214	36
	Bilateral	117	166	29

### Transcranial direct current stimulation

Direct current was transmitted by a saline-soaked pair of surface sponges (35 cm^2^) and delivered by a battery-driven, constant current stimulator with a maximum output of 10 mA (NeuroConn; http://www.neuroconn.de/). Two hundred sixty-five patients received tDCS with cathodal electrode placed over the left DLPFC and the anode placed overlying the right DLPFC, 380 patients received tDCS based on EEG connectivity. Patients who received tDCS were randomly assigned to DLPFC tDCS or tDCS based on EEG. In addition 65 received no tDCS, and were used as a waiting list control group.

For the EEG driven tDCS, EEGs (Mitsar, Saint Petersburg, Russia) were obtained 1 week before the tACS stimulation in a fully lighted room with each participant sitting upright in a comfortable chair. The EEG was sampled with 19 electrodes (Fp1, Fp2, F7, F3, Fz, F4, F8, T7, C3, Cz, C4, T8, P7, P3, Pz, P4, P8, O1 O2) in the standard 10–20 International placements referenced to linked lobes and impedances were checked to remain below 5 kΩ. Data were collected for 100 2-s epochs eyes closed, sampling rate = 1024 Hz, and band passed 0.15–200 Hz. Data were resampled to 128 Hz, band-pass filtered (fast Fourier transform filter) to 2–44 Hz. These data were transposed into Eureka! Software (Congedo, 2002), plotted and carefully inspected for manual artifact rejection. All episodic artifacts including eye blinks, eye movements, teeth clenching, body movement, or ECG artifacts were removed from the stream of the EEG.

### Target localization

To determine the precise location of the gamma band functional connectivity, i.e., lagged phase synchronization is used. This was operationally defined as the brain area retrieved on source localized EEG, using sLORETA software, in each individual, which has most connectivity lines in the gamma band (30–45 Hz). This measure is threshold invariant (when increasing the threshold the amount of functional connections will decrease in all areas, but the area with most connections will remain the area with most functional connections) and clinically applicable. The brain area with the highest gamma band functional connectivity was elected as the target for cathode placement. The area for placing the anode was determined by the highest theta band functional connectivity.

Connectivity can be calculated by analyzing phase synchronization or coherence. However, any measure of dependence is highly contaminated with an instantaneous, non-physiological contribution due to volume conduction (Pascual-Marqui, 2007a). Therefore, Pascual-Marqui, (Pascual-Marqui, 2007b) introduced a new technique (i.e., Hermitian covariance matrices) that removes this confounding factor. As such, this measure of dependence can be applied to any number of brain areas jointly, i.e., distributed cortical networks, whose activity can be estimated with sLORETA. Measures of linear dependence (coherence) between the multivariate time series are defined. The measures are expressed as the sum of lagged dependence and instantaneous dependence. The measures are non-negative, and take the value zero only when there is independence and are defined in the gamma (30.5–45 Hz) frequency domain. Thus only the lagged phase synchronization is used. Regions of interest were defined based on previous brain research on tinnitus (see Table 2 for overview). Based on the functional connectivity analysis the region which forms a hub (i.e., ROI that is connected with the most ROI) is selected as the target area to be stimulated.

**Table 2:** **Regions of interest**.

Brodmann area	Brain area	Author
BA6	Supplementary motor area	Jastreboff (1990)
BA7	Precuneus	Heller (2003)
BA9-46	Dorsolateral prefontal cortex	Heller (2003), Axelsson and Ringdahl (1989), Baguley (2002), Eggermont and Roberts (2004), Smits et al. (2007), van der Loo et al. (2009)
BA10	Frontopolar cortex	Vanneste et al. (2010a), Muhlnickel et al. (1998)
BA11	Orbitofrontal	Vanneste et al. (2010a)
BA13	Insula	Heller (2003), Weisz et al. (2007)
BA21-22	Secondary auditory cortex	Lockwood et al. (1999), Langguth et al. (2006), Muhlnickel et al. (1998)
BA23-31	Posterior cingulate cortex	Heller (2003)
BA24-32	Dorsal anterior cingulate cortex	Heller (2003), Smits et al. (2007)
BA25	Subgenual anterior cingulate cortex	Heller (2003), Smits et al. (2007)
BA39-40	Angular gyrus	Muhlnickel et al. (1998)
BA41-42	Primary auditory cortex	Lockwood et al. (1999), Langguth et al. (2006), Muhlnickel et al. (1998)

### Evaluation

A visual analog scale for tinnitus intensity (“How loud is your tinnitus?: 0 = no tinnitus and 10 = as loud as imaginable”) and tinnitus distress (“How stressful is your tinnitus? 0 = no distress and 10 = suicidal distress”) was asked before (pre) and directly after (post) tDCS stimulation. The responses were collected by the person who applied the tDCS.

### Statistical analyses

A three-stage analysis was performed. First the overall results were calculated to verify whether there was an effect obtained by tDCS in comparison to baseline. This is followed by a second analysis evaluating whether there was a difference between frontal and EEG driven tDCS. This is then followed by a third analysis looking at the response rate and response size differences between bifrontal and EEG driven tDCS. Calculations were performed using SPSS 18.0 software package.

#### Overall effects

A repeated measure ANOVA was conducted with VAS distress and VAS intensity pre-tDCS and post tDCS as within-subjects variables and condition (frontal tDCS, EEG driven tDCS, and waiting list) as between-subjects variable. To verify that the within-variables were normally distributed a Kolmogorov–Smirnov test was applied. This demonstrated that the within-variables did not deviate from a normal distribution. In addition we reported the effect size by including the partial eta squared (η^2^). The standards for these effect sizes are small (η^2^ = 0.01), medium (η^2^ = 0.06), and large (η^2^ = 0.14).

#### A comparison between the effects obtained for frontal and EEG driven tDCS

A second repeated measures ANOVA was conducted with the obtained difference (Pre – Post tDCS) as within-subjects variable and group (Frontal tDCS versus EEG driven tDCS) as between-subjects variable to verify whether there was a significant difference in the obtained suppression on both distress and intensity. In addition we also reported the effect size by including the partial eta squared (η^2^).

#### The effects for responders only

We applied a logistic regression with condition (including frontal tDCS and EEG driven tDCS) as independent variable and responding (No = 0 or Yes = 1) as dependent variable. Responders are defined as patients who obtain minimally 10% suppression, while non-responders are defined as those patients obtain less than 10% improvement. A repeated measures ANOVA was conducted with VAS distress and VAS intensity pre-tDCS and post tDCS as within-subjects variables and condition (frontal tDCS and EEG driven tDCS) as between group variable to verify if there was a significant difference for the responders only on both distress and intensity. We also reported the effect size by including the partial eta squared (η^2^).

## Results

### Overall effects

A comparison between the baseline measurements between the three different groups revealed no significant effect for both tinnitus distress (*F* = 0.24, *p* = 0.79) and tinnitus intensity (*F* = 2.01, *p* = 0.13).

**Figure 1:**
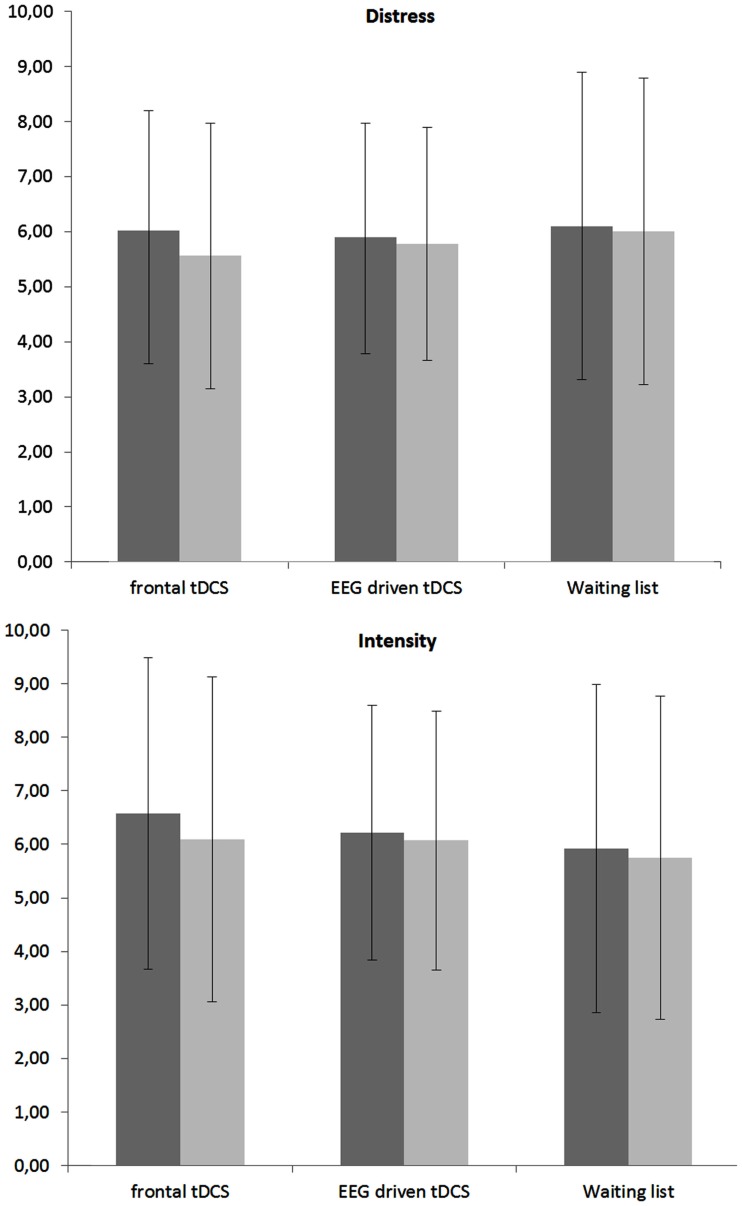
**Mean VAS score pre and post tDCS (within-subjects variable) for respectively frontal tDCS, EEG driven tDCS, and a Waiting list group (between-subjects variable) for both tinnitus distress and tinnitus intensity**.

A repeated measures ANOVA revealed a significant main effect for pre versus post measurement (*F* = 17.19, *p* < 0.001, η^2^ = 0.05). A closer look to the data indicated that for distress (*F* = 25.01, *p* < 0.001, η^2^ = 0.03) there was a significant decrease in the post tDCS in comparison to pre-tDCS. For intensity a similar effect was obtained (*F* = 31.16, *p* < 0.001, η^2^ = 0.04) demonstrating there was a significant decrease in the post tDCS in comparison to pre-tDCS. No significant main effect was obtained for condition (frontal tDCS, EEG driven tDCS, and waiting list) on both tinnitus distress and tinnitus intensity. In addition an interaction effect was obtained between condition (frontal tDCS, EEG driven tDCS, and waiting list) × tDCS (pre versus post) for both tinnitus distress and tinnitus intensity (*F* = 6.59, *p* < 0.001, η^2^ = 0.02). For tinnitus distress it was shown that both frontal tDCS (*F* = 68.73, *p* < 0.001, η^2^ = 0.07) and EEG driven tDCS (*F* = 7.42, *p* < 0.01, η^2^ = 0.04) had post tDCS a significant reduction in comparison to pre-tDCS scores (see Figure 1). No effect was obtained for the waiting list group. For tinnitus intensity also a significant decrease was demonstrated post tDCS in comparison to pre-tDCS scores for respectively frontal tDCS (*F* = 69.95, *p* < 0.001,η^2^ = 0.09) and EEG driven tDCS (*F* = 9.39, *p* < 0.01, η^2^ = 0.01). Again, no effect was obtained for the waiting list group. Figure 1 gives an overview of the obtained results.

### A comparison between the effects obtained for frontal and EEG driven tDCS

**Figure 2:**
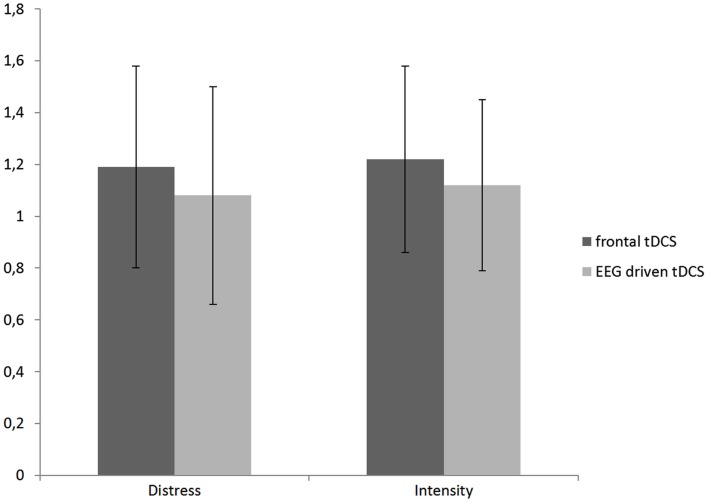
**A comparison between the obtained difference (Pre – Post tDCS) for the distress and intensity measurement in respectively frontal and EEG driven tDCS**.

Further analysis indicated a difference for the bifrontal tDCS group for respectively tinnitus distress (*F* = 17.72, *p* < 0.001, η^2^ = 0.02) and tinnitus intensity (*F* = 10.74, *p* < 0.01, η^2^ = 0.01). See Figure 2 for an overview.

### The effects for responders only

If we look what patients respond, we found that bifrontal tDCS in comparison to EEG driven tDCS had a larger response rate for both tinnitus distress (χ^2^ = 17.03, *p* < 0.001, β = -0.99) and tinnitus intensity (χ^2^ = 10.41, *p* < 0.01, β = -0.68). That is, for tinnitus distress 19.2% responded to bifrontal tDCS in comparison to 8.2% for EEG driven tDCS and for tinnitus intensity 22.1% responded to bifrontal tDCS in comparison to 12.5% for EEG driven tDCS.

A repeated measures ANOVA including only the responders on both distress and intensity revealed a significant main effect for pre versus post measurement (*F* = 115.22, *p* < 0.001, η^2^ = 0.13), with a decrease on distress of 36.74% and on intensity of 28.22%. No significant main effect was obtained for condition (frontal tDCS, and EEG driven tDCS) as well for the interaction effect between condition (frontal tDCS, and EEG driven tDCS) × tDCS (pre versus post) for both.

## Discussion

The main surprising result of the study is that EEG driven placement of anode and cathode does not benefit tinnitus suppression rates in comparison to bifrontal tDCS with anode overlying the right DLPFC and the cathode overlying the left DLPFC. Even though theoretically one would expect that EEG driven tDCS should be superior to “blind” bifrontal stimulation, this does not seem to be case. Multiple explanations can be proposed.

A first explanation is related to the parameter used. Possibly gamma lagged phase synchronization is not a good parameter to determine where to place the cathode. It has indeed been shown, both in the visual system (Antal et al., 2004) and the DLPFC for tinnitus suppression (Vanneste et al., 2011b) that gamma band activity in the area under the cathode is decreased and increased in the area under the anode. However, it is yet unknown whether this also means that gamma functional connectivity as measured by lagged phase synchronization is also modulated. As tDCS brings neurons closer or further away from threshold depending on the polarity, this should not automatically lead to changes in phase synchronization.

A second possible explanation is that even though gamma band activity is important in tinnitus perception, it has also been proposed that this gamma band activity only leads to conscious perception if this activity is connected to a larger network involved in conscious perception (van der Loo et al., 2009; De Ridder et al., 2011a). Gamma band activity, which normally waxes and wanes, and is spatially restricted to small areas, actually is nested on low frequency activity, predominantly theta activity, in order to connect to widespread larger networks, both for normal cognition (Canolty et al., 2006; Lisman and Buzsaki, 2008) and in tinnitus (De Ridder et al., 2011a). In a recent study it has been demonstrated that auditory attention control is mediated via gamma band activity in different brain areas, which were connected via theta activity, the phase of which determined gamma synchronization (Doesburg et al., 2012). Thus it could have been better to select theta connectivity as a potential prognostic biomarker, as it is possible theta is a carrier wave on which the information rich gamma activity is nested.

Another explanation can be related to the exactness of the electrode positioning. As gamma band activity is usually spatially restricted and only present in a small focal area, the exact positioning of the cathode and anode might be critically involved in the success of the tDCS stimulation. Since this study was not performed using neuronavigation, because of methodological and technical reasons [(1) sLORETA uses standard head model, (2) EEG cannot be read into the neuronavigation machine] it cannot be excluded that the electrodes were not spatially correctly positioned (Vanneste et al., 2011b).

Thus, whereas the results of the bifrontal tDCS seem to confirm previous studies (Vanneste et al., 2010c, 2011b; Faber et al., 2011; Frank et al., 2012), in that bifrontal tDCS with anode overlying the right DLPFC and the cathode the left DLPFC has a beneficial effect on tinnitus loudness and distress perception, the use of gamma band functional connectivity seems not bring any advantage to tDCS for tinnitus suppression. Using other potential biomarkers, such as gamma band activity, or theta functional connectivity could theoretically be better alternatives. Further studies will have to elucidate whether brain state based tDCS has any advantages over “blind” bifrontal stimulation.

## Conflict of Interest Statement

The authors declare that the research was conducted in the absence of any commercial or financial relationships that could be construed as a potential conflict of interest.
